# Lack of Renal Tubular Glucocorticoid Receptor Decreases the Thiazide-Sensitive Na^+^/Cl^–^ Cotransporter NCC and Transiently Affects Sodium Handling

**DOI:** 10.3389/fphys.2019.00989

**Published:** 2019-08-14

**Authors:** Jérémie Canonica, Simona Frateschi, Emilie Boscardin, Anna Ebering, Chloé Sergi, Yannick Jäger, Thibaud Peyrollaz, Anne-Marie Mérillat, Marc Maillard, Petra Klusonova, Alex Odermatt, Robert Koesters, Anne Debonneville, Olivier Staub, Sophia N. Verouti, Edith Hummler

**Affiliations:** ^1^Department of Pharmacology and Toxicology, University of Lausanne, Lausanne, Switzerland; ^2^National Centre of Competence in Research “Kidney.CH”, Lausanne, Switzerland; ^3^Department of Nephrology Service, University Hospital of Lausanne (CHUV), Lausanne, Switzerland; ^4^Department of Pharmaceutical Sciences, University of Basel, Basel, Switzerland; ^5^Hôpital Tenon, Université Pierre et Marie Curie, Paris, France

**Keywords:** glucocorticoid receptor, sodium transport, sodium and hydrogen exchanger 3, Na^+^-K^+^-Cl^–^ cotransporter, thiazide-sensitive Na^+^/Cl^–^ cotransporter, blood pressure, blood pressure dipping

## Abstract

Chronic glucocorticoid infusion impairs NCC activity and induces a non-dipping profile in mice, suggesting that glucocorticoids are essential for daily blood pressure variations. In this paper, we studied mice lacking the renal tubular glucocorticoid receptor (GR) in adulthood (GR knockouts, *Nr3c1*^*Pax8/LC1*^). Upon standard salt diet, *Nr3c1*^*Pax8/LC1*^ mice grow normally, but show reduced NCC activity despite normal plasma aldosterone levels. Following diet switch to low sodium, *Nr3c1*^*Pax8/LC1*^ mice exhibit a transient but significant reduction in the activity of NCC and expression of NHE3 and NKCC2 accompanied by significant increased Spak activity. This is followed by transiently increased urinary sodium excretion and higher plasma aldosterone concentrations. Plasma corticosterone levels and 11βHSD2 mRNA expression and activity in the whole kidney remain unchanged. High salt diet does not affect whole body Na^+^ and/or K^+^ balance and NCC activity is not reduced, but leads to a significant increase in diastolic blood pressure dipping in *Nr3c1*^*Pax8/LC1*^ mice. When high sodium treatment is followed by 48 h of darkness, NCC abundance is reduced in knockout mice although activity is not different. Our data show that upon Na^+^ restriction renal tubular GR-deficiency transiently affects Na^+^ handling and transport pathways. Overall, upon standard, low Na^+^ and high Na^+^ diet exposure Na^+^ and K^+^ balance is maintained as evidenced by normal plasma and urinary Na^+^ and K^+^ and aldosterone concentrations.

## Introduction

The glucocorticoid receptor (GR, *Nr3c1*) belongs to the same nuclear steroid receptor family as the mineralocorticoid receptor (MR, *Nr3c2*) and acts as ligand-dependent transcription factor. It binds corticosteroid hormones like cortisol (human) and corticosterone (rodents) (Lu et al., 2006). GR is expressed along the whole nephron where it overlaps with the expression of MR in the aldosterone-sensitive distal nephron (ASDN), defined by the expression of the enzyme 11β-hydroxysteroid dehydrogenase type 2 (11β-HSD2) (Ackermann et al., 2010). Circulating aldosterone, cortisol and corticosterone can activate both receptors with different affinity. 11β-HSD2 inactivates 11β-hydroxy glucocorticoids and thereby protects MR from illicit activation by glucocorticoids (Valinsky et al., 2018). Ackermann et al. (2010) proposed that ligand-induced nuclear translocation of both steroid receptors may be part of a segment- and cell type-specific regulation in the kidney, as a differential nuclear translocation was observed upon corticosteroid treatment along the rat nephron.

There is *in vitro* evidence that glucocorticoids stimulate renal sodium transport, which thus finally mediates mineralocorticoid-like effects (Naray-Fejes-Toth and Fejes-Toth, 1990; Schmidt et al., 1993; Bens et al., 1999). Gaeggeler et al. (2005) established a quantitative relationship between MR and GR occupancy and sodium transport response in a mouse cortical collecting duct cell line. Under stress conditions, when free cortisol (or corticosterone) reaches high levels, amiloride-sensitive sodium transport is stimulated despite intact 11β-HSD2 expression or activity, possibly as a result of 11β-HSD2 saturation (Odermatt et al., 2001). Adrenocorticotropic hormone excess promotes renal sodium reabsorption contributing to the increased blood pressure, and both GR and MR pathways are involved (Bailey et al., 2009). Persistent increase of circulating glucocorticoids is clinically relevant and may contribute to the pathogenesis of hypertension (Bailey, 2017). In mice, elevated hormone levels promote a nocturnal hypertension and induce a non-dipping blood pressure profile that is restored by thiazides (Ivy et al., 2016). Finally, glucocorticoid treatment transiently changes the renal clock gene transcription and thus influences peripheral clocks, although the underlying mechanism is not yet completely defined (Balsalobre et al., 2000; Sujino et al., 2012).

Studies on the implication of GR in renal sodium transport have been limited because of the early lethality of the total GR knockout mainly due to respiratory failure (Cole et al., 1995). Mice conditionally over-expressing the human GR within the cortical collecting duct maintain normal sodium absorption and blood pressure suggesting tubular compensation (Nguyen Dinh Cat et al., 2009). Mice with constitutive partial knockout of GR in the distal nephron using the *Ksp:Cre* line exhibit mildly elevated baseline blood pressure levels, and they show a similar hypertensive response to dexamethasone (Goodwin et al., 2010). Ivy and coworkers recently reported that mice with global GR haploinsufficiency exhibit increased blood pressure that is further increased by high salt diet. In this model, corticosterone, and to a lesser extent deoxycorticosterone excretion, was increased in mutant mice following a high-salt challenge, that may lead to systemic effects (Ivy et al., 2018).

Until now, the role of renal tubular GR on electrolyte handling and blood pressure control remained unknown. In this study, we contribute with an inducible, tubular-specific GR knockout model to models of systemic GR antagonism, global haploinsufficiency or non-inducible GR deletion. Using low, moderate and high Na^+^-containing diets, we analyzed mice carrying a kidney-tubule specific knockout of GR. Our results demonstrate that lack of GR expression in renal tubules affects the abundance and activity of some sodium transporters, in particular NCC, but maintains sodium and potassium balance. Na^+^ restriction transiently induces downregulation of several sodium transport systems with a transient effect on Na^+^ handling.

## Results

### Upon Standard Sodium Diet Na^+^ Balance Is Not Affected in *Nr3c1*^*Pax8/LC1*^ Mice Despite Decreased NCC Activity

**FIGURE 1:**
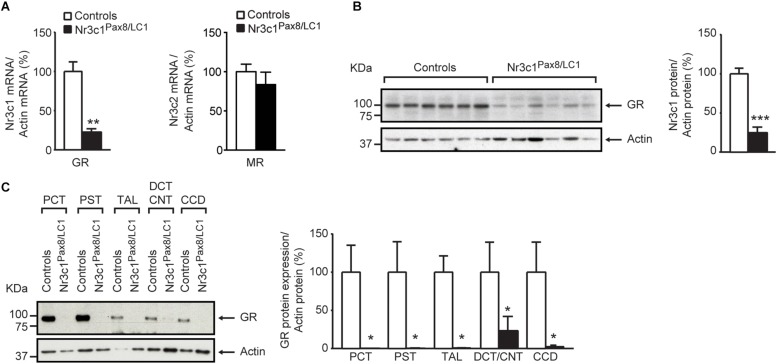
Glucocorticoid receptor (GR) is efficiently deleted in the kidney of adult *Nr3c1*^*Pax8/LC1*^ knockout mice. **(A)** Quantification of GR (encoded by *Nr3c1*, *n* = 4 per genotype; left panel) and MR (encoded by Nr3c2, *n* = 5 per genotype; right panel) mRNA expression relative to β-actin in the whole kidneys of *Nr3c1*^*Pax8/LC1*^ KO mice and their control littermates (*n* = 4 per genotype, unpaired two-tailed *t*-test). **(B)** Representative Western blot analysis of GR (encoded by *Nr3c1*) and β-actin in whole kidney lysates under a regular sodium diet (*n* = 6 per genotype) and its quantification. Samples were collected at 8–10 a.m. local time and analyzed by unpaired two-tailed *t*-test. **(C)** Representative analysis of GR protein expression by Western blot analysis and its quantification from (*n* = 3) micro-dissected renal tubules. β-actin was used as loading control. PCT proximal convoluted tubule, PST proximal straight tubule, TAL thick ascending limb, DCT distal convoluted tubule, CNT connecting tubule, CCD cortical collecting duct. Values are means ± SEM, and datasets analyzed by unpaired two-tailed *t*-test. *^∗^P* < 0.05, *^∗∗^P* < 0.01, *^∗∗∗^P* < 0.001.

**FIGURE 2:**
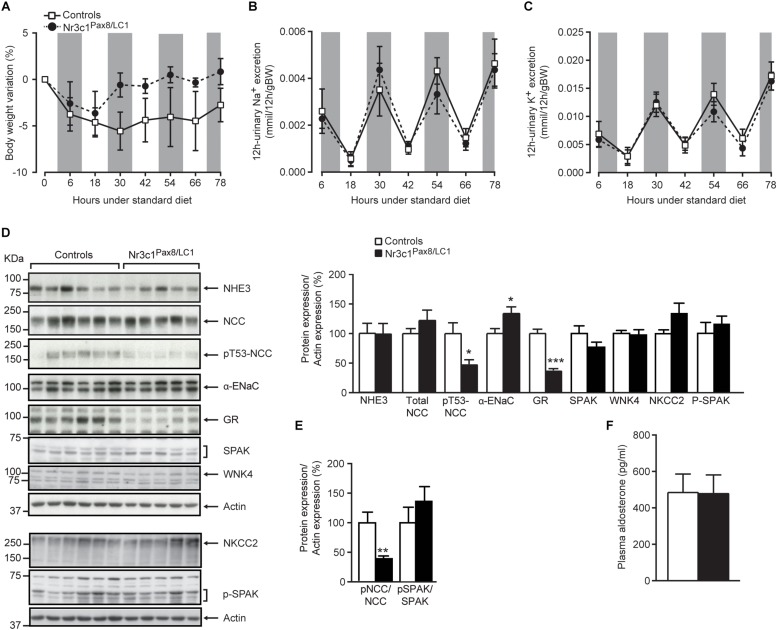
*Nr3c1*^*Pax8/LC1*^ knockouts display normal sodium and potassium balance, but altered sodium transporting protein expression under a normal salt diet. **(A)** Body weight change (expressed as percent of initial body weight), **(B)** 12 h-urinary sodium and **(C)** potassium excretion in *Nr3c1*^*Pax8/LC1*^ knockout and control mice. All parameters were determined each 12 h in metabolic cages following 2 weeks of doxycycline treatment, and under a normal salt diet. Values are means ± SEM, and datasets were analyzed by ANOVA. The gray zone indicates the active night period (light off) and the white zone indicates the inactive day period (light on). **(D)** Western blot analysis of NHE3, NKCC2, NCC, pT53-NCC, αENaC, Spak, pSpak, Wnk4 and GR on kidney lysates from control and *Nr3c1*^*Pax8/LC1*^ knockout mice under regular sodium diet. β-actin was used as loading control. Samples were collected at the end of the metabolic cage experiment (end of active night phase, 8–10 a.m. local time), and the quantification of Western blots (right panel), and **(E)** the ratio of pNCC/NCC and pSpak/Spak protein abundance. **(F)** Plasma aldosterone levels in control and *Nr3c1*^*Pax8/LC1*^ knockout mice under normal sodium diet. Values are means ± SEM; *^∗^P* < 0.05, *^∗∗^P* < 0.01, *^∗∗∗^P* < 0.001. *n* = 6 controls (3 males and 3 females) and *n* = 5 knockouts (2 females and 3 males). Datasets were analyzed by unpaired two-tailed *t*-test **(D,F)**.

Following 2 weeks of doxycycline treatment, the tubular GR was efficiently deleted in *Nr3c1*^*Pax8/LC1*^ knockout mice. Whole kidney lysates from *Nr3c1*^*Pax8/LC1*^ mice exhibited an about 80% reduced Nr3c1 mRNA transcript and protein expression (Figure 1A, left panel and Figure 1B), and tubular microdissection revealed a near-complete absence of Nr3c1 protein in all tubular segments with the exception of the DCT/CNT which showed a significantly reduced Nr3c1 expression (Figure 1C and Supplementary Figures S1A,B). In contrast to GR, MR (*Nr3c2*) mRNA expression was not affected in the whole kidney of *Nr3c1*^*Pax8/LC1*^ mutant mice (Figure 1A, right panel). Hepatic GR protein expression in Nr3c1^*Pax8/LC1*^ mice did not significantly change when mice on standard diet were analyzed 2 weeks, 2 months or 3 months following doxycycline treatment (Supplementary Figures S1C–E). Upon doxycycline induction and standard diet (Supplementary Figure S2A), *Nr3c1*^*Pax8/LC1*^ mice survived, grew normally and did not present any significant change in body weight, urinary Na^+^ and K^+^ excretion (Figures 2A–C). Lack of renal GR did not change the protein abundance of sodium transporters like NHE3 or NCC. However, phosphoT53-NCC (pNCC) as well as the ratio pNCC/NCC were drastically decreased (Figures 2D,E). The protein expression of the alpha subunit of ENaC was significantly increased (Figure 2D), although plasma aldosterone levels did not differ between the *Nr3c1* control and knockout mice (Figure 2D). Further physiological parameters as food and water intake, feces output, urine volume, Na^+^ and K^+^ intake, and plasma Na^+^ and K^+^ concentrations were not different between the two groups (Supplementary Figure S3). Overall, mice lacking the renal GR survive well and show no obvious kidney phenotype. However, missing GR signaling in the kidney affects αENaC abundance and NCC activity. We thus addressed whether the *Nr3c1*^*Pax8/LC1*^ mice are able to adapt to the change from normal to sodium-deficient diet.

### *Nr3c1*^*Pax8/LC1*^ Mice Exhibit a Transient Na^+^ Wasting on Na^+^-Deprivation

**FIGURE 3:**
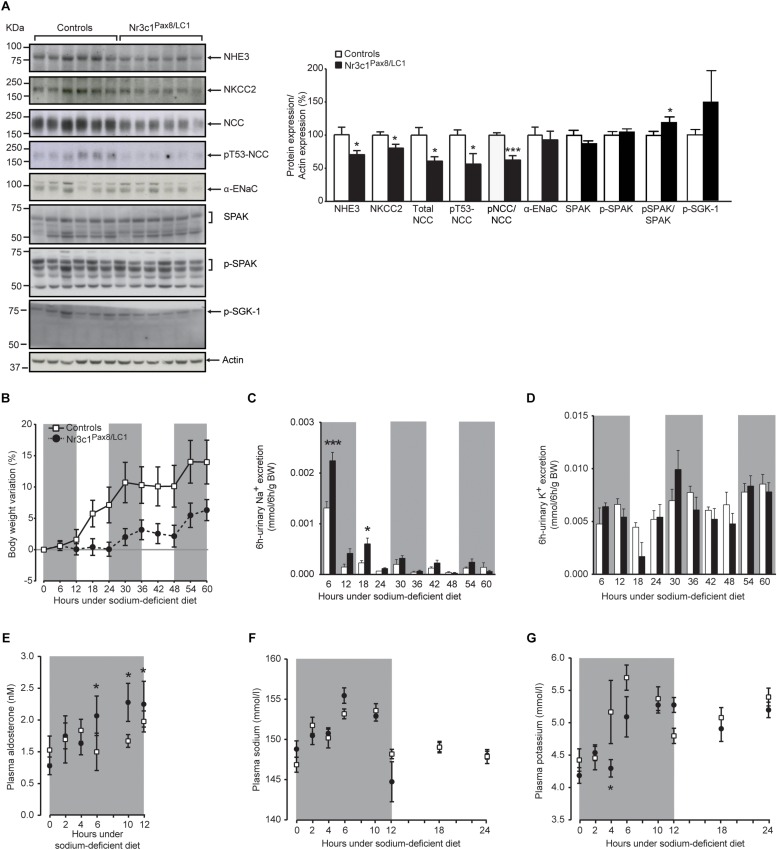
*Nr3c1*^*Pax8/LC1*^ knockouts transiently display altered sodium and potassium balance accompanied by higher plasma aldosterone level upon switching to low sodium diet. **(A)** Western blot analysis of NHE3, NKCC2, NCC, phosphorylated NCC (pT53-NCC), αENaC, Spak, pSpak, and pSgk1 on kidney lysates from control and *Nr3c1*^*Pax8/LC1*^ knockout mice (*n* = 6 per genotype) 10 h after the switch from regular to sodium-deficient diet at 4 a.m. local time (active night phase). β-actin was used as loading control. Graph shows quantification of Western blots. Values are means ± SEM, datasets were analyzed by unpaired two-tailed *t*-test. **(B)** Body weight changes (expressed as percent of initial body weight) in *Nr3c1*^*Pax8/LC1*^ KO and control mice. **(C)** 6 h urinary sodium and **(D)** potassium excretion determined in *Nr3c1*^*Pax8/LC1*^ KO (*n* = 5, male animals) and control mice (*n* = 7, male animals). Time points 6 and 18 correspond to 12 p.m. and 12 a.m. local time, respectively. **(E)** Plasma aldosterone levels in control and *Nr3c1*^*Pax8/LC1*^ knockout mice (*n* = 7–10, per genotype, all males) following switch from regular to sodium-deficient diet. Time points 6, 10 and 12 correspond to 12 p.m., 4 and 6 a.m. local time, respectively. **(F)** Plasma sodium and **(G)** plasma potassium concentrations in *Nr3c1*^*Pax8/LC1*^ KO and control mice measured at different time points following low sodium treatment (*n* = 6–8 per genotype, all males). All parameters were determined each 6 h in metabolic cages 3 months after 2 weeks of doxycycline treatment, and following the switch from regular to sodium-deficient diet. The gray zone indicates the active night period (light off) and the white zone indicates the inactive day period (light on). Values are means ± SEM and datasets were analyzed by ANOVA (**B–G**; compared to control mice, *^∗^P* < 0.05, *^∗∗∗^P* < 0.001).

Low Na^+^ diet transiently decreased NCC activity although the expression of the phosphorylated form of Sgk-1 (pSgk-1) did not differ (Figure 3A). This was accompanied by an increased plasma aldosterone concentration at 6, 10 and 12 h (Figure 3E). We followed the physiological parameters as well as the expression of sodium transporting proteins at time points 0, 4, 6, 10, 12, and 36 h following diet switch from a normal to a sodium-deprived diet (Supplementary Figure S2B). At 10 h following diet switch, *Nr3c1*^*Pax8/LC1*^ mice showed a significant reduction in the sodium transporting proteins NHE3, NKCC2, NCC and its phosphorylated T53 form (Figure 3 and Supplementary Figures S4A,B). The pNKCC2 abundance does not differ (data not shown). *Nr3c1*^*Pax8/LC1*^ did not display a significant difference in body weight gain compared to their littermate controls (Figure 3B). Reduced Na^+^-transporting protein abundance was accompanied by a significantly increased urinary Na^+^ excretion within 6 h and between 12-18 h following diet switch (Figure 3C). Yet, similar 6 h -urinary K^+^ excretion, food intake, water intake, urine volume, urine volume to water intake ratio were observed in the two different groups (Figures 3D–G and Supplementary Figures S4C–F). At 4 h following diet switch, plasma potassium, but not plasma sodium, was significantly decreased in the *Nr3c1* knockout mice (Figures 3F,G). We found a high variation of values for this time point which could be due to varying individual food intake. No change was observed for urinary Na^+^/creatinine and K^+^/creatinine and urinary Na^+^/Na^+^ intake and K^+^/K^+^ intake, plasma corticosterone levels, the ratio of urinary aldosterone to creatinine concentration, 11βHSD2 mRNA expression and activity, and Wnk4 mRNA levels in the whole kidney (Supplementary Figure S5).

### Upon High Salt Diet Nr3c1^*Pax*8/*LC*1^ Mice Present Increased Diastolic Blood Pressure Dipping

To test whether mice lacking the renal GR signaling are more sensitive to high salt, *Nr3c1*^*Pax8/LC1*^ experimental and control mice were switched from a standard to high sodium diet and monitored in metabolic cages every 12 h for four consecutive days (Supplementary Figure S2C). *Nr3c1*^*Pax8/LC1*^ mice did not differ from the controls with respect to body weight, urinary Na^+^ and K^+^ excretion, food and water intake, feces output, urine volume, Na^+^ and K^+^ intake, and plasma Na^+^ and K^+^ concentrations and renal sodium and potassium transporters abundance (Supplementary Figure S6). When high Na^+^ treatment was prolonged up to 23 days and followed by 48 h of continuous darkness to challenge GR-dependent effects in the circadian rhythm (Supplementary Figure S2D), there was no further marked kidney phenotype with the exception of significant differences in the knockout mice at specific time points for food, Na^+^ and K^+^ intake (Supplementary Figure S7). At the end of the dark phase, we observed significant decrease of total NCC abundance (Supplementary Figure S7F). The plasma Na^+^ and K^+^ concentrations (Supplementary Figures S7G,H) and protein expression levels of NHE3, NKCC2, pT53-NCC and αENaC did not differ (Supplementary Figure S7F), as well as Na^+^ and K^+^ excretion, water intake and body weight gain (Supplementary Figures S7I–L).

**FIGURE 4:**
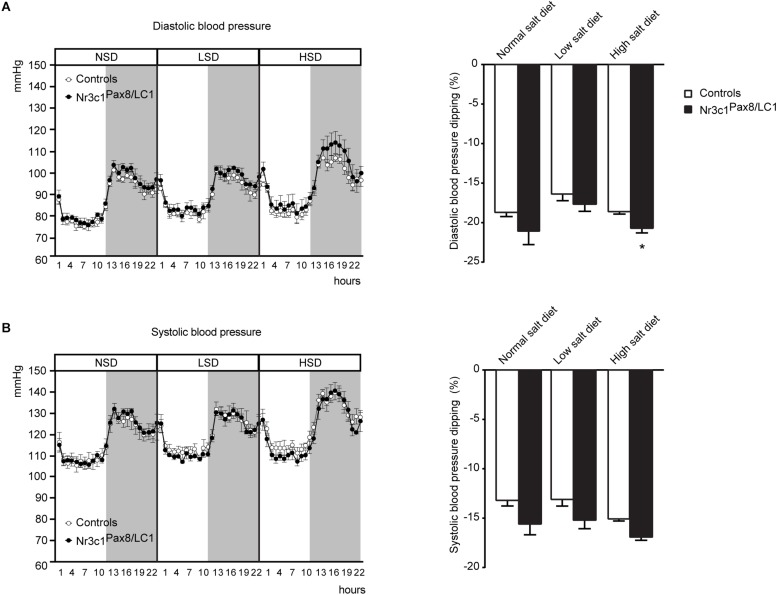
*Nr3c1*^*Pax8/LC1*^ knockout mice present increased diastolic blood pressure dipping upon switch to high salt diet. **(A)** Diastolic and **(B)** systolic blood pressure in control and *Nr3c1*^*Pax8/LC1*^ KO mice. For each mouse, a mean of 10 day recordings (normal sodium diet, NSD), 6 day recordings (low sodium diet, LSD) and 5-day recordings (high sodium diet, HSD) is presented; then, the mean of these values was determined for each genotype. Data are mean ± SEM (*n* = 4 male animals per genotype). Histograms display the mean of differences ± SEM between percent decline in diurnal diastolic and systolic blood pressure under normal, low salt and high salt diet. The gray zone indicates 12 h active night period (light off) and the white zone indicates the 12 h inactive day period (light on). Datasets were analyzed by unpaired two-tailed *t*-test. *^∗^P* < 0.05.

Finally, we assessed the consequences of the shift from normal (10 days) to low Na^+^ diet (6 days), and then to high Na^+^ diet (5 days) on diastolic and systolic blood pressure using telemetry (Supplementary Figure S2D). Upon all diet conditions, diastolic and systolic blood pressure did not differ (Figure 4). We observed a significant increase in diastolic blood pressure dipping in the knockout group under a high salt treatment (Figure 4A, right panel). Systolic blood pressure dipping did not present significant difference among the different genotypes (Figure 4B, right panel).

In summary, mice lacking the renal tubular GR do not show alteration in their whole Na^+^ or K^+^ balance despite changes in Na^+^ transporting proteins. Our results unveil a transient role of the renal glucocorticoid receptor in Na^+^ handling.

## Discussion

### *Nr3c1*^*Pax8/LC1*^ Knockout Mice Overall Maintain Na^+^ and K^+^ Balance but Show a Transient Salt-Losing Phenotype Upon Na^+^ Restriction

Previous *in vitro* and *in vivo* studies proposed a role of GR in renal MR-mediated Na^+^ transport in a compensatory and/or cooperative manner (Berger et al., 1998; Gaeggeler et al., 2005; Hunter et al., 2014; Canonica et al., 2016; Terker et al., 2016; Ivy et al., 2018), although it could not yet be functionally confirmed. In this study, we used adult inducible nephron-specific GR knockout (*Nr3c1*^*Pax8/LC1*^) mice to explore the consequences of renal tubular GR deletion for Na^+^ handling and blood pressure regulation. This allowed us to discriminate between systemic and renal-specific effects of GR signaling in tubular cells. *Nr3c1*^*Pax8/LC1*^ mice with lacking GR in renal tubular cells (Figure 1 and Supplementary Figure S1) show no obvious phenotype (Figure 2 and Supplementary Figure S3) contrarily to the MR (Nr3c2^*Pax8/LC1*^) knockout mice which developed severe pseudohypoaldosteronism type 1 (PHA-1) under standard Na^+^ diet that worsened upon Na^+^-deficient diet (Canonica et al., 2016; Terker et al., 2016).

*Nr3c1*^*Pax8/LC1*^ mice maintained overall Na^+^ and K^+^ balance under a standard salt diet, as evidenced by normal plasma aldosterone and Na^+^ and K^+^ concentrations. Though, this was accompanied by decreased phosphorylated NCC under standard (Figure 2) and transiently decreased total NCC expression and phosphorylation under low sodium diet (Figure 3), as well as the reduced total NCC abundance following high Na^+^ and prolonged dark phase exposure (Supplementary Figure S7). Decreased NCC expression and/or activity in the *Nr3c1*^*Pax8/LC1*^ knockouts might be an attempt to maintain or even increase Na^+^ delivery via ENaC in the CNT and CD. However, we found neither a hyperkalemic nor a hypovolemic stimulus, nor signs of increased renal MR mRNA expression, nor increased plasma aldosterone levels (Figure 1A, right panel, Figures 2B,C,E). Interestingly, a decrease in NCC and pNCC expression was also documented in MR knockout mice under standard, low and high Na^+^ diet (Canonica et al., 2016). This might reflect a compensation to maintain normal Na^+^ and K^+^ balance in the context of the aldosterone paradox (Arroyo et al., 2011) although low Na^+^ diet exposure would normally increase pNCC expression (Chiga et al., 2008). In this context, it is worth mentioning that the data of Mu et al. (2011) suggested a key role for GR mediating β-adrenergic stimulation on the activity of NCC. The NCC downregulation in *Nr3c1*^*Pax8/LC1*^ knockout mice despite normal circulating aldosterone and corticosterone levels is thus consistent with the lack of renal GR signaling in our study (Figure 2) or following adrenalectomy (Ivy et al., 2016). Additionally, maintenance of normal fluid volume and blood pressure homeostasis is thought to be regulated through altered NCC and ENaC activity (Mu et al., 2011), which might explain increased αENaC subunit abundance in the absence of altered aldosterone levels (Figure 2). However, ENaC and NKCC2 activity depend further on e.g., their phosphorylation and surface expression.

Interestingly, these GR knockout mice develop hyperkalemia upon K^+^ restriction (Keppner et al., unpublished), underlining the role of NCC as key player in K^+^ homeostasis (Wang et al., 2018). Upon Na^+^ restriction, *Nr3c1*^*Pax8/LC1*^ knockout mice transiently increase urinary sodium loss and plasma aldosterone concentration. Thus absence of renal GR may prevent the dual occupancy of MR and GR to induce optimal Na^+^ reabsorption during acute Na^+^-deprivation. This is accompanied by transient but significant downregulation of Na^+^ transporting proteins (Figure 3). GR might be partly required for aldosterone-induced and MR-dependent signaling as GR/MR heterodimer formation and/or protein–protein interaction with GR as co-factor for MR homodimers were reported previously in human neuronal cells (de Kloet et al., 2005; Tsugita et al., 2009) and a mouse cortical collecting duct cell line (Gaeggeler et al., 2005). Bergann et al. (2011) found that GR was indispensable for aldosterone- and MR-dependent ENaC induction in a human colonic cell line. In this context, daily injection of the glucocorticoid betamethasone prolonged the survival of global MR knockouts but could not avoid further lethality (Berger et al., 1998), indicating that GR signaling only partly substitute renal MR-mediated Na^+^ absorption.

### High Salt Diet Treatment Increases Diastolic Blood Pressure Dipping, but Maintains Na^+^ and K^+^ Balance in GR Knockout Mice

We monitored blood pressure under standard, low and high Na^+^ diet in *Nr3c1*^*Pax8/LC1*^ knockout and control littermates, and found that the knockouts although not significantly different, tend to an overall higher blood pressure, especially following sodium load (Figure 4). In agreement, Goodwin and coworkers previously studied mice with a tubular-specific knockout of GR in the distal nephron using the Ksp-Cre line and found mildly elevated baseline blood pressure (Goodwin et al., 2010). It was however, not reported whether plasma glucocorticoid levels were increased. In absence of renal GR, several consecutive days of high Na^+^ diet leads to significant increased blood pressure dipping despite normal NCC activity (Figure 4 and Supplementary Figure S7L). Upon glucocorticoid excess following corticosterone infusion and thus GR stimulation, Ivy and coworkers reported that NCC was inappropriately upregulated leading to a non-dipping blood pressure profile (Ivy et al., 2016). In *Nr3c1*^*Pax8/LC1*^ knockout mice, NCC is downregulated upon standard Na^+^ diet indeed identifying NCC as a molecular target of renal tubular GR-mediated signaling in the absence of glucocorticoid excess.

Since GR has been previously linked to circadian clock function (Yang, 2010), we tested whether high Na^+^ diet plus prolonged dark phase affect Na^+^ and K^+^ handling. As previously reported, peripheral clocks in kidney are regulated and reset by a daily stimulus of corticosterone administration (Balsalobre et al., 2000; Sujino et al., 2012). However, we cannot find evidence of a disturbed circadian clock function. High Na^+^ diet combined with prolonged dark phase significantly reduces NCC protein abundance (Supplementary Figure S7) that is not observed following high Na^+^ treatment alone (Supplementary Figure S6). Overall, Na^+^ and K^+^ balance is maintained (Supplementary Figure S7) indicating that renal GR may only play a minor role in the renal circadian rhythm. Interestingly, the knockout of the renal molecular circadian clock did not reveal obvious changes in renal Na^+^, K^+^ or water handling (Nikolaeva et al., 2016) suggesting a complex compensatory mechanism that still needs future investigations.

In summary, the present renal tubular-specific GR knockout model allows to study the consequences of GR-deficiency without higher circulating glucocorticoid level. Here, we demonstrate an implication although transiently of the renal tubular GR in NCC regulation and Na^+^ handling. Even upon different low and high Na^+^ challenges, GR knockout maintain their Na^+^ and K^+^ balance, and their blood pressure.

## Materials and Methods

### Animals

Mice were housed in ventilated cages at a constant temperature (23 ± 1°C) and humidity (60%) with an automatic 12 h light/dark cycle. Water and laboratory chow were supplied *ad libitum*. Unless differently stated, data originated from 3 to 6-week-old animals (males and females) carrying the Ren-1^c^ allele.

### Generation of Inducible Renal Tubule-Specific *Nr3c1*^*Pax8/LC1*^ KO Mice

Mice lacking GR along the nephron and in the collecting duct system of the kidney were obtained by using the floxed GR mouse line (*Nr3c1*^*lox/lox*^) (Tronche et al., 1999), and the Pax8:rtTA^*tg/0*^;TRE:LC-1^*tg/0*^ transgenic animals (Schonig et al., 2002; Traykova-Brauch et al., 2008). Inducible renal tubule-specific knockouts (*Nr3c1^*lox/lox*^;Pax8:rtTA^*tg/0*^; TRE:LC-1^*tg/0*^; Nr3c1^*Pax8/LC1*^*), and littermate controls (*Nr3c1^*lox/lox*^;Pax8:rtTA^*tg/0*^* and *Nr3c1^*lox/lox*^;TRE:LC-1^*tg/0*^*) were obtained by interbreeding *Nr3c1^*lox/lox*^; Pax8:rtTA^*tg/0*^* with *Nr3c1^*lox/lox*^;TRE:LC-1^*tg/0*^* double transgenic mice. Nr3c1 deletion was induced by doxycycline hydrochloride treatment (Sigma, Deisenhofen, Germany; 2 mg/ml and 2% sucrose in drinking water) (Plachov et al., 1990; Poleev et al., 1992; Traykova-Brauch et al., 2008) for 15 days in 25 day-old control and experimental mice, unless differentially stated. Animals were fed a standard salt diet (0.17% Na^+^ and 0.97% K^+^; Supplementary Figure S2A), or a short-term low sodium diet (0.01% Na^+^ and 0.97% K^+^, Supplementary Figure S2B) for 4 days in metabolic cages. Furthermore, animals were subjected to a short term high salt diet (3.5% Na^+^ and 0.97% K^+^, Supplementary Figure S2C) for 6 days, or a long term high salt diet for 20 days (Supplementary Figure S2D). For blood pressure measurements, male mice underwent surgery after doxycycline treatment followed by 10 days recovery and blood pressure recording for ten consecutive days (normal/standard salt), 6 days low salt and 5 days high salt diet (Supplementary Figure S2E).

### Genotyping

DNA was extracted from ear and kidney biopsies. PCR analysis was performed by using the following primers: Pax8-rtTA: ST1 sense (5′-CCATGTCTAGACTGGACAAGA-3′); ST2 antisense (5′-CTCCAGGCCACATATGATTAG-3′); LC-1: Cre3 sense (5′-TCGCTGCATTACCGGTCGATGC-3′); Cre4 antisense (5′- CCATGAGTGAACGAACCTGGTCG-3′); Myogenin: 50S sense (5′-TTACGTCCATCGTGGACAGC-3′); 51S antisense (5′-TGG GCTGGGTGTTAGTCTTA-3′); Renin: for (5′-CCTACACAC TCAGCAGTACGGA-3′); rev (5′-GAACTTGCGGATGAAGGT GGCA-3′); Myogenin amplification served as a control for DNA integrity. Thermocycling conditions for a routine PCR to amplify the *Pax8:rtTA*, *TRE:LC*-*1* and the myogenin amplicons consisted of 37 cycles. Denaturation, annealing and extension were carried out during 1 min at 94, 56, and 72°C, respectively. Floxed *Nr3c1* alleles were detected by PCR on whole kidney using the following primers: GR/*Nr3c1*: GRflox-1 sense (5′-GG CATGCACATTACTGGCCTTCT-3′); GRflox-2 antisense (5′-CCTTCTCATTCCATGTCAGCATGT-3′); GRflox-3 antisense (5′-GTGTAGCAGCCAGCTTACAGGA-3′). Thermocycling conditions for a routine PCR to amplify the floxed allele consisted of 35 cycles. Denaturation, annealing and extension were carried at 95, 63, and 72°C during 30 s, 1 min and 1 min, respectively.

### Quantitative RT-PCR on Kidney Samples

Kidneys were isolated, frozen in liquid nitrogen and stored at –80°C. A Tissue-Lyser machine (Qiagen, Hilden, Germany) was used to homogenize the tissue. RNA was extracted from the lysed tissue using the guanidnium thiocyanate-phenol-chloroform extraction method (QIAzol lysis reagent, Qiagen, Hilden, Germany) and its concentration and quality were assessed by the Nano Drop (Witec Ag ND-1000 Spectrophotometer). cDNA was synthetized by using the PrimeScript RT reagent Kit (Takara Bio, Inc., Japan). Real-time PCRs were performed by Taqman^®^ PCR (Applied Biosystems 7500, Foster City, CA, United States). Primers and probe mixtures (Mm00433833_mH for GR; Mm01251104_m1 for 11β-HSD2; Mn02342887_mH for Ren-1^c^; 4352341E for β-actin) and the Taqman Gene Expression Master Mix were purchased and used according to the manufacturer’s instructions (Applied Biosystem, Foster City, CA, United States). Each measurement was performed in duplicate. Quantification of fluorescence was performed by calculating the ΔΔC_T_ values.

### Western Blot Analysis

Freshly isolated kidneys were homogenized using the polytron. Homogenates were centrifuged for 10 min at 4°C at 11000 rpm. Protein concentration was measured by the Bradford method. Proteins were loaded and separated on 10% polyacrylamide gels by SDS-PAGE, subjected to a constant electric current of 25 mA. Proteins were then transferred onto a PVDF (Perkin Elmer, Boston, MA, United States) or nitrocellulose membrane (Amersham Hybond-ECL, GE Healthcare) applying a constant current of 100 V during 3 h. Membranes were subsequently blotted for Nr3c1 (GR, 1:1000; Santa Cruz, Dallas, TX, United States), Scnn1a (Sorensen et al., 2013) (1:500), Slc12a3 (Sorensen et al., 2013) (1:500), pT53-Slc12a3 (Sorensen et al., 2013) (1:1000), Slc12a1 (Kaplan et al., 1996) (1:10000), Slc9a3 (Wiederkehr et al., 2001) (1:10), Spak (Millipore, 07–2271; 1:100), pSpak (Millipore, 07-2273; 1:1000), pNKCC2 (Picard et al., 2014); 1:200), Wnk4 (1/250; Abcam, Switzerland), pSgk1 (1/500; Santa Cruz, 16744) and β-Actin (1:1000; Sigma-Aldrich, Buchs, Switzerland). Anti-rabbit IgG (1:10000; Amersham, Burkinghampshire, United Kingdom), anti-mouse IgG (1:10000; Jackson Immuno Research, Baltimore, PA, United States) and anti-goat (Santa Cruz; 1:10000) secondary antibody were coupled with the horseradish Peroxidase (GE Healthcare, millipore) and the ECL reagents (GE Healthcare or Pierce, Rockford, IL, United States). Membranes were exposed on a photographic film (GE Healthcare, Millipore) in a cassette (Axon Lab) and developed. Band intensity was measured by using the Image Studio Lite Software from LI-COR Biosciences.

### Kidney Perfusion and Microdissection

Mice were anesthetized by a mixture of Ketamine/Xylazine/Acepromazine (100 mg/kg/15 mg/kg/2.5 mg/kg) injected intraperitoneally. Renal artery perfusion was performed into the renal artery by using a catheter (10 ml of DMEM F-12, Dulbecco’s Modified Eagle Medium: Nutrient Mixture F-12 followed by 10 ml of liberase 0.9 mg/ml, Liberase Blendzyme 4, Hoffmann-La Roche, Inc.). Then, kidneys were micro-dissected as previously described (Christensen et al., 2010). 2 cm of each segment (PC, PS, TAL, DCT/CNT and CD) were recovered and processed for *Nr3c1* (encoding GR) and β*-actin*. The microdissections were performed on 3 experimental and 3 control animals.

### Metabolic Cage Studies

Control and knockout mice from same litter were individually placed into mouse metabolic cages (Tecniplast, Buguggiate, Italy) during 6 days and fed with different salt diets (normal sodium 0.17%, sodium-deficient 0.02% and high sodium 3.5% diet, ssniff Spezialdiäten GmbH, Soest, Germany). During the experimentation, body weight, urine volume, water and food consumption were measured every 12 h at the end of the active night phase (6 a.m. local time) and at the end of the resting day phase (6 p.m. local time), for normal and high sodium diets. For the low sodium diet, metabolic parameters were measured each 6 h (time 0 and time 12 corresponding to 6 p.m. and 6 a.m. local time, respectively). The animals had free access to food and water. At the end of the experiment, mice were sacrificed and organs and blood were collected for further analyses. Supplementary Figure S2 details the used protocols. Each experiment was validated for GR expression by Western blot: only experiments showing at least a 50% GR reduction on total kidney lysates were considered for renal GR deletion. Unless indicated, samples collection was performed at the beginning of the resting light phase (8–10 a.m. local time).

### Urine and Plasma Analysis

Urine samples (6–24 h) were collected in metabolic cages. Blood samples were recovered at the end of experiment by retro-orbital bleeding, and the procedure was terminal. Urinary and plasma sodium and potassium concentrations were measured by using the IL943 Flame Photometer (Instrumentation Laboratory, United Kingdom). Plasma aldosterone levels were measured by using the Coat-A-Count RIA kit (Siemens Medical Solutions Diagnostics, Ballerup, Denmark). Plasma corticosterone levels were quantified by ultra-high performance liquid chromatography tandem mass spectrometry as previously described (Seibert et al., 2014).

### Determination of 11β-HSD2 Enzyme Activity

Kidneys were pulverized in liquid nitrogen and resuspended in buffer TS2 (100 mM NaCl, 1 mM EGTA, 1 mM EDTA, 1 mM MgCl2, 250 mM sucrose, and 20 mM Tris–HCl at pH 7.4). Enzyme activity was measured by adding 250 μM NAD + and 100 nM of tritium-labeled corticosterone and incubation for 10 min at 37°C. Reactions were terminated by adding unlabeled corticosterone and 11-dehydrocorticosterone in methanol, followed by separation of steroids by thin layer chromatography determination of product formation investigated by scintillation counting as previously described (Schweizer et al., 2004; Balazs et al., 2008).

### Blood Pressure Measurements

Blood pressure measurements were performed in conscious unrestrained male mice by using Data Science International (DSI) telemetry system. Mice were treated with doxycycline during 2 weeks and fed with a normal salt diet. Mice were allowed to recover for 10 days after the implantation of telemetry device before starting the blood pressure recording. The blood pressure recording was carried out during 10 days under a normal salt diet, then during 6 days under a low sodium diet, and finally during 5 days under a high sodium diet (Supplementary Figure S2E). Resting phase (day) blood pressure dipping was computed by calculating the difference between day and night blood pressures, expressed in percentage of night, for systolic and diastolic blood pressures.

### Statistical Analysis

Measurements were analyzed with GraphPad Prism using ANOVA for repeated measurements and unpaired two-tailed Student’s *t*-test. Blood pressure measurements were analyzed with R by evaluating the area under the curve during the 12 h night active phase and the 12 h day resting phase, and comparing control and knockout group by using the *t*-test. Data are presented as mean ± SEM. Differences displaying a *P*-value smaller than 0.05 were considered as statistically significant. ^∗^*P* < 0.05, ^∗∗^*P* < 0.01, ^∗∗∗^*P* < 0.001. Sample size was computed by using G power (effect size *d* = 1.94, α error = 0.05, power = 0.9, ratio N1/N2 = 1: sample size group 1 = 7, sample size group 2 = 7).

## Data Availability

All datasets analyzed for this study are included in the manuscript and/or the Supplementary Files.

## Ethics Statement

Animal maintenance and experimental procedures were performed in accordance with the Swiss federal guidelines and approved by the local veterinarian authorities (Direction générale de l’ agriculture, de la viticulture et des affaires vétérinaires) of the Canton de Vaud, Switzerland.

## Author Contributions

JC, EB, CS, SF, YJ, TP, A-MM, SV, AE, MM, PK, and AD performed the experiments. RK, AO, and OS designed the research. SF and EH wrote the manuscript. EH, JC, SF, and SV designed and supervised the study. All authors revised the manuscript and read and approved the submitted version.

## Conflict of Interest Statement

The authors declare that the research was conducted in the absence of any commercial or financial relationships that could be construed as a potential conflict of interest.

## References

[B1] Ackermann D. Gresko N. Carrel M. Loffing-Cueni D. Habermehl D. Gomez-Sanchez C. (2010). In vivo nuclear translocation of mineralocorticoid and glucocorticoid receptors in rat kidney: differential effect of corticosteroids along the distal tubule. *Am. J. Physiol. Renal. Physiol.* 299 F1473–F1485. 10.1152/ajprenal.00437.2010 20861076

[B2] Arroyo J. P. Ronzaud C. Lagnaz D. Staub O. Gamba G. (2011). Aldosterone paradox: differential regulation of ion transport in distal nephron. *Physiology* 26 115–123. 10.1152/physiol.00049.2010 21487030

[B3] Bailey M. A. (2017). 11beta-Hydroxysteroid dehydrogenases and hypertension in the metabolic syndrome. *Curr. Hypertens. Rep.* 19:100. 10.1007/s11906-017-0797-z 29138984 PMC5686277

[B4] Bailey M. A. Mullins J. J. Kenyon C. J. (2009). Mineralocorticoid and glucocorticoid receptors stimulate epithelial sodium channel activity in a mouse model of Cushing syndrome. *Hypertension* 54 890–896. 10.1161/HYPERTENSIONAHA.109.134973 19635986

[B5] Balazs Z. Schweizer R. A. Frey F. J. Rohner-Jeanrenaud F. Odermatt A. (2008). DHEA induces 11 -HSD2 by acting on CCAAT/enhancer-binding proteins. *J. Am. Soc. Nephrol.* 19 92–101. 10.1681/asn.2007030263 18032797 PMC2391033

[B6] Balsalobre A. Brown S. A. Marcacci L. Tronche F. Kellendonk C. Reichardt H. M. (2000). Resetting of circadian time in peripheral tissues by glucocorticoid signaling. *Science* 289 2344–2347. 10.1126/science.289.5488.2344 11009419

[B7] Bens M. Vallet V. Cluzeaud F. Pascual-Letallec L. Kahn A. Rafestin-Oblin M. E. (1999). Corticosteroid-dependent sodium transport in a novel immortalized mouse collecting duct principal cell line. *J. Am. Soc. Nephrol.* 10 923–934. 10232677 10.1681/ASN.V105923

[B8] Bergann T. Fromm A. Borden S. A. Fromm M. Schulzke J. D. (2011). Glucocorticoid receptor is indispensable for physiological responses to aldosterone in epithelial Na+ channel induction via the mineralocorticoid receptor in a human colonic cell line. *Eur. J. Cell Biol.* 90 432–439. 10.1016/j.ejcb.2011.01.001 21354648

[B9] Berger S. Bleich M. Schmid W. Cole T. J. Peters J. Watanabe H. (1998). Mineralocorticoid receptor knockout mice: pathophysiology of Na+ metabolism. *Proc. Natl. Acad. Sci. U.S.A.* 95 9424–9429. 10.1073/pnas.95.16.9424 9689096 PMC21354

[B10] Canonica J. Sergi C. Maillard M. Klusonova P. Odermatt A. Koesters R. (2016). Adult nephron-specific MR-deficient mice develop a severe renal PHA-1 phenotype. *Pflugers Arch.* 468 895–908. 10.1007/s00424-015-1785-2 26762397

[B11] Chiga M. Rai T. Yang S. S. Ohta A. Takizawa T. Sasaki S. (2008). Dietary salt regulates the phosphorylation of OSR1/SPAK kinases and the sodium chloride cotransporter through aldosterone. *Kidney Int.* 74 1403–1409. 10.1038/ki.2008.451 18800028

[B12] Christensen B. M. Perrier R. Wang Q. Zuber A. M. Maillard M. Mordasini D. (2010). Sodium and potassium balance depends on alphaENaC expression in connecting tubule. *J. Am. Soc. Nephrol.* 21 1942–1951. 10.1681/ASN.2009101077 20947633 PMC3014008

[B13] Cole T. J. Blendy J. A. Monaghan A. P. Krieglstein K. Schmid W. Aguzzi A. (1995). Targeted disruption of the glucocorticoid receptor gene blocks adrenergic chromaffin cell development and severely retards lung maturation. *Genes Dev.* 9 1608–1621. 10.1101/gad.9.13.1608 7628695

[B14] de Kloet E. R. Joels M. Holsboer F. (2005). Stress and the brain: from adaptation to disease. *Nat. Rev. Neurosci.* 6 463–475. 10.1038/nrn1683 15891777

[B15] Gaeggeler H. P. Gonzalez-Rodriguez E. Jaeger N. F. Loffing-Cueni D. Norregaard R. Loffing J. (2005). Mineralocorticoid versus glucocorticoid receptor occupancy mediating aldosterone-stimulated sodium transport in a novel renal cell line. *J. Am. Soc. Nephrol.* 16 878–891. 10.1681/asn.2004121110 15743993

[B16] Goodwin J. E. Zhang J. Velazquez H. Geller D. S. (2010). The glucocorticoid receptor in the distal nephron is not necessary for the development or maintenance of dexamethasone-induced hypertension. *Biochem. Biophys. Res. Commun.* 394 266–271. 10.1016/j.bbrc.2010.02.123 20188070 PMC2946623

[B17] Hunter R. W. Ivy J. R. Bailey M. A. (2014). Glucocorticoids and renal Na+ transport: implications for hypertension and salt sensitivity. *J. Physiol.* 592 1731–1744. 10.1113/jphysiol.2013.267609 24535442 PMC4001748

[B18] Ivy J. R. Evans L. C. Moorhouse R. Richardson R. V. Al-Dujaili E. A. S. Flatman P. W. (2018). Renal and blood pressure response to a high-salt diet in mice with reduced global expression of the glucocorticoid receptor. *Front. Physiol.* 9:848. 10.3389/fphys.2018.00848 30038578 PMC6046455

[B19] Ivy J. R. Oosthuyzen W. Peltz T. S. Howarth A. R. Hunter R. W. Dhaun N. (2016). Glucocorticoids induce nondipping blood pressure by activating the thiazide-sensitive cotransporter. *Hypertension* 67 1029–1037. 10.1161/HYPERTENSIONAHA.115.06977 26953322 PMC4905621

[B20] Kaplan M. R. Plotkin M. D. Lee W. S. Xu Z. C. Lytton J. Hebert S. C. (1996). Apical localization of the Na-K-Cl cotransporter, rBSC1, on rat thick ascending limbs. *Kidney Int.* 49 40–47. 10.1038/ki.1996.6 8770947

[B21] Lu N. Z. Wardell S. E. Burnstein K. L. Defranco D. Fuller P. J. Giguere V. (2006). International union of pharmacology. LXV. the pharmacology and classification of the nuclear receptor superfamily: glucocorticoid, mineralocorticoid, progesterone, and androgen receptors. *Pharmacol. Rev.* 58 782–797. 10.1124/pr.58.4.9 17132855

[B22] Mu S. Shimosawa T. Ogura S. Wang H. Uetake Y. Kawakami-Mori F. (2011). Epigenetic modulation of the renal beta-adrenergic-WNK4 pathway in salt-sensitive hypertension. *Nat. Med.* 17 573–580. 10.1038/nm.2337 21499270

[B23] Naray-Fejes-Toth A. Fejes-Toth G. (1990). Glucocorticoid receptors mediate mineralocorticoid-like effects in cultured collecting duct cells. *Am. J. Physiol.* 259(4 Pt 2), F672–F678. 2221105 10.1152/ajprenal.1990.259.4.F672

[B24] Nguyen Dinh Cat A. Ouvrard-Pascaud A. Tronche F. Clemessy M. Gonzalez-Nunez D. Farman N. (2009). Conditional transgenic mice for studying the role of the glucocorticoid receptor in the renal collecting duct. *Endocrinology* 150 2202–2210. 10.1210/en.2008-1531 19106216

[B25] Nikolaeva S. Ansermet C. Centeno G. Pradervand S. Bize V. Mordasini D. (2016). Nephron-specific deletion of circadian clock gene bmal1 alters the plasma and renal metabolome and impairs drug disposition. *J. Am. Soc. Nephrol.* 27 2997–3004. 10.1681/asn.2015091055 27056296 PMC5042670

[B26] Odermatt A. Arnold P. Frey F. J. (2001). The intracellular localization of the mineralocorticoid receptor is regulated by 11beta-hydroxysteroid dehydrogenase type 2. *J. Biol. Chem.* 276 28484–28492. 10.1074/jbc.m100374200 11350956

[B27] Picard N. Trompf K. Yang C. L. Miller R. L. Carrel M. Loffing-Cueni D. (2014). Protein phosphatase 1 inhibitor-1 deficiency reduces phosphorylation of renal NaCl cotransporter and causes arterial hypotension. *J. Am. Soc. Nephrol.* 25 511–522. 10.1681/ASN.2012121202 24231659 PMC3935578

[B28] Plachov D. Chowdhury K. Walther C. Simon D. Guenet J. L. Gruss P. (1990). Pax8, a murine paired box gene expressed in the developing excretory system and thyroid gland. *Development* 110 643–651. 1723950 10.1242/dev.110.2.643

[B29] Poleev A. Fickenscher H. Mundlos S. Winterpacht A. Zabel B. Fidler A. (1992). PAX8, a human paired box gene: isolation and expression in developing thyroid, kidney and Wilms’ tumors. *Development* 116 611–623. 1337742 10.1242/dev.116.3.611

[B30] Schmidt T. J. Husted R. F. Stokes J. B. (1993). Steroid hormone stimulation of Na+ transport in A6 cells is mediated via glucocorticoid receptors. *Am. J. Physiol.* 264(4 Pt 1), C875–C884. 8476020 10.1152/ajpcell.1993.264.4.C875

[B31] Schonig K. Schwenk F. Rajewsky K. Bujard H. (2002). Stringent doxycycline dependent control of CRE recombinase in vivo. *Nucleic Acids Res.* 30:e134. 12466566 10.1093/nar/gnf134 PMC137989

[B32] Schweizer R. A. Zurcher M. Balazs Z. Dick B. Odermatt A. (2004). Rapid hepatic metabolism of 7-ketocholesterol by 11beta-hydroxysteroid dehydrogenase type 1: species-specific differences between the rat, human, and hamster enzyme. *J. Biol. Chem.* 279 18415–18424. 10.1074/jbc.m313615200 14973125

[B33] Seibert J. Hysek C. M. Penno C. A. Schmid Y. Kratschmar D. V. Liechti M. E. (2014). Acute effects of 3,4-methylenedioxymethamphetamine and methylphenidate on circulating steroid levels in healthy subjects. *Neuroendocrinology* 100 17–25. 10.1159/000364879 24903002

[B34] Sorensen M. V. Grossmann S. Roesinger M. Gresko N. Todkar A. P. Barmettler G. (2013). Rapid dephosphorylation of the renal sodium chloride cotransporter in response to oral potassium intake in mice. *Kidney Int.* 83 811–824. 10.1038/ki.2013.14 23447069

[B35] Sujino M. Furukawa K. Koinuma S. Fujioka A. Nagano M. Iigo M. (2012). Differential entrainment of peripheral clocks in the rat by glucocorticoid and feeding. *Endocrinology* 153 2277–2286. 10.1210/en.2011-1794 22434077

[B36] Terker A. S. Yarbrough B. Ferdaus M. Z. Lazelle R. A. Erspamer K. J. Meermeier N. P. (2016). “Direct and indirect mineralocorticoid Effects determine distal salt transport”. *J. Am. Soc. Nephrol.* 27 2436–2445. 10.1681/ASN.2015070815 26712527 PMC4978056

[B37] Traykova-Brauch M. Schonig K. Greiner O. Miloud T. Jauch A. Bode M. (2008). An efficient and versatile system for acute and chronic modulation of renal tubular function in transgenic mice. *Nat. Med.* 14 979–984. 10.1038/nm.1865 18724376 PMC3446847

[B38] Tronche F. Kellendonk C. Kretz O. Gass P. Anlag K. Orban P. C. (1999). Disruption of the glucocorticoid receptor gene in the nervous system results in reduced anxiety. *Nat. Genet.* 23 99–103. 10.1038/12703 10471508

[B39] Tsugita M. Iwasaki Y. Nishiyama M. Taguchi T. Shinahara M. Taniguchi Y. (2009). Glucocorticoid receptor plays an indispensable role in mineralocorticoid receptor-dependent transcription in GR-deficient BE(2)C and T84 cells in vitro. *Mol. Cell Endocrinol.* 302 18–25. 10.1016/j.mce.2008.12.008 19146914

[B40] Valinsky W. C. Touyz R. M. Shrier A. (2018). Aldosterone, SGK1, and ion channels in the kidney. *Clin. Sci.* 132 173–183. 10.1042/CS20171525 29352074 PMC5817097

[B41] Wang M. X. Cuevas C. A. Su X. T. Wu P. Gao Z. X. Lin D. H. (2018). Potassium intake modulates the thiazide-sensitive sodium-chloride cotransporter (NCC) activity via the Kir4.1 potassium channel. *Kidney Int.* 93 893–902. 10.1016/j.kint.2017.10.023 29310825 PMC6481177

[B42] Wiederkehr M. R. Di Sole F. Collazo R. Quinones H. Fan L. Murer H. (2001). Characterization of acute inhibition of Na/H exchanger NHE-3 by dopamine in opossum kidney cells. *Kidney Int.* 59 197–209. 10.1046/j.1523-1755.2001.00480.x 11135072

[B43] Yang X. (2010). A wheel of time: the circadian clock, nuclear receptors, and physiology. *Genes Dev.* 24 741–747. 10.1101/gad.1920710 20395363 PMC2854389

