# Supplementary material for: Lack of Renal Tubular Glucocorticoid Receptor Decreases the Thiazide-Sensitive Na+/Cl– Cotransporter NCC and Transiently Affects Sodium Handling
Source: Front Physiol. 2019 Aug 14;10:989. doi: 10.3389/fphys.2019.00989 (PMC6702950; doi:10.3389/fphys.2019.00989)
Supplement: Supplementary file 1 [file Table_1.DOCX]

**SUPPLEMENTARY FIGURES**


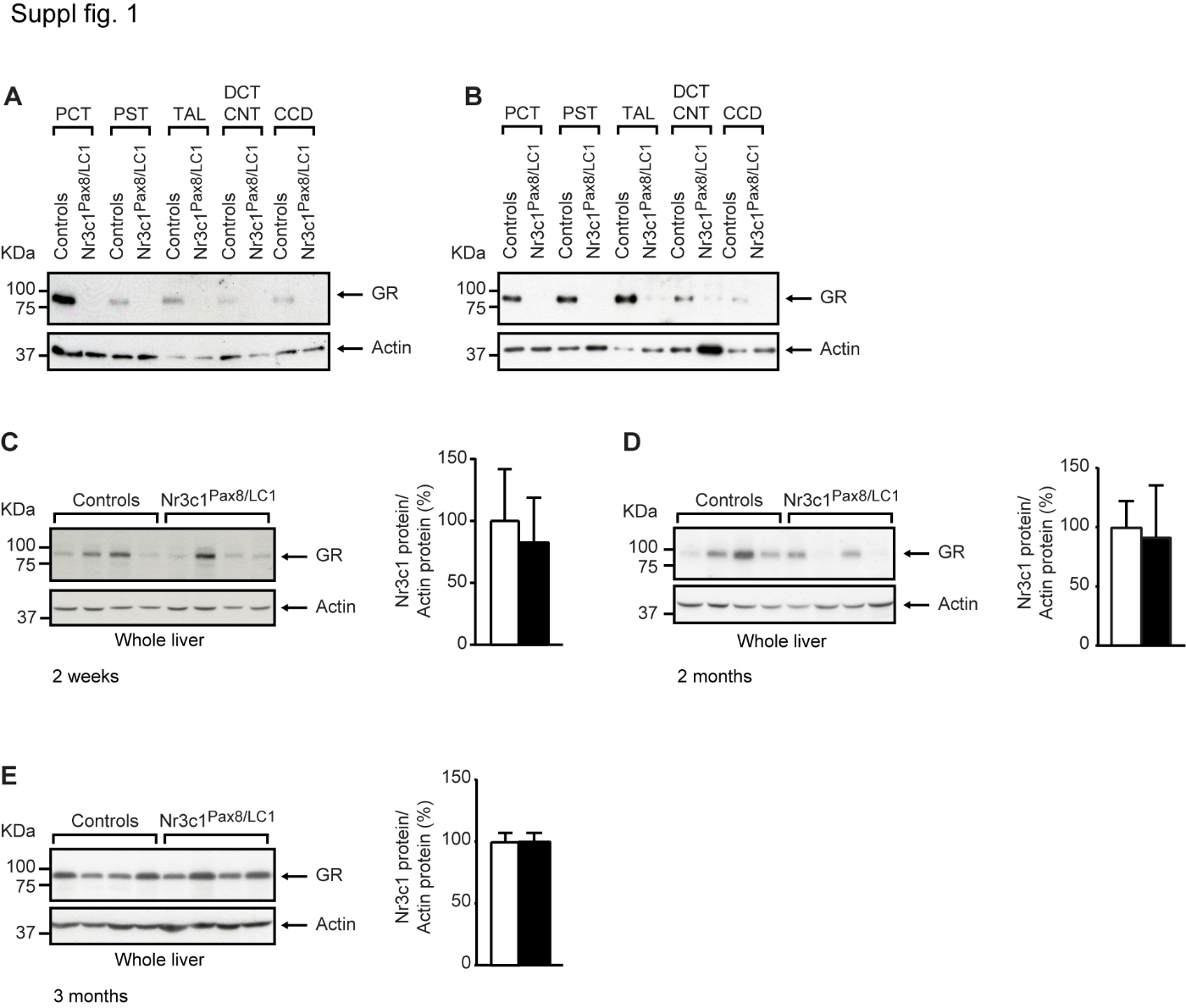


**Figure S1 Validation of the knockout model** (**A** and **B**) Western blot analyses of microdissected renal tubules from 2 controls and 2 Nr3c1^Pax8/LC1^ knockout mice. β-actin was used as loading control. PCT proximal convoluted tubule, PST proximal straight tubule, TAL thick ascending limb, DCT distal convoluted tubule, CNT connecting tubule, CCD cortical collecting duct. (**C**) Western blot analysis of GR and its quantification in whole liver lysates from controls and Nr3c1^Pax8/LC1^ knockout mice straight after and (**D**) 2 months and (**E**) 3 months following doxycycline induction under a standard salt diet. Histograms represent quantification of the blots on the left. *n* = 4 per genotype, all males. Values are means ± SEM. Samples were collected at the end of active night phase (8-9 a.m. local time). Datasets were analyzed by unpaired two-tailed t test.


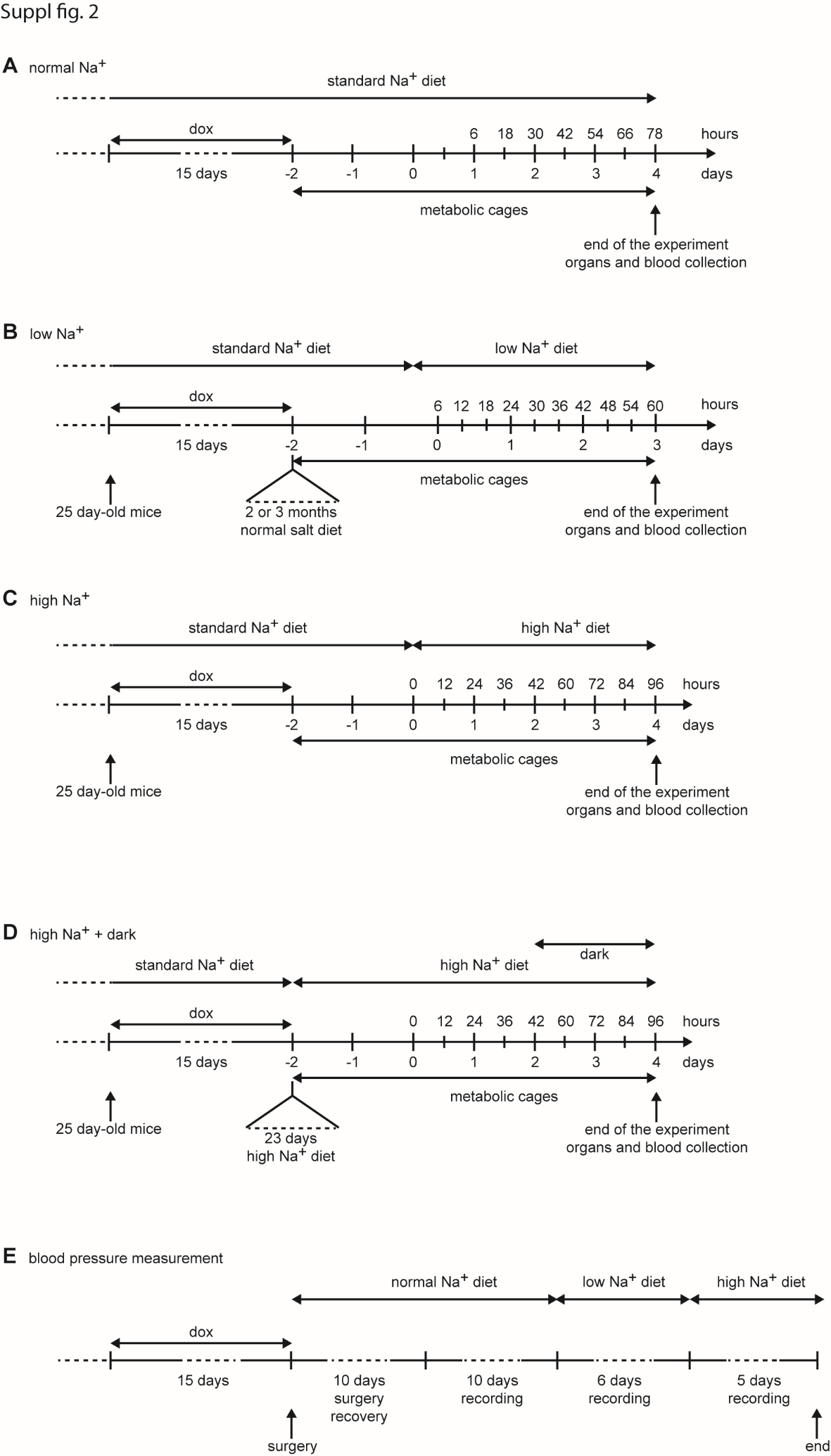


**Figure S2 Graphical representations of dietary protocols.** Nr3c1 control and experimental (Nr3c1^Pax8/LC1^, knockout) mice were induced at the age of 25 days after birth with doxycycline under standard salt diet (0.17% Na^+^ and 0.97% K^+^). Measurements were performed each 12 hours for normal and high salt treatments at the end of the active (night) and active (light) period, and each 6 hours for low sodium treatment. Animals adapted to the metabolic cage environment during two days before the measurements. (**A**) Normal sodium: metabolic cage study on standard salt diet 0.17% Na^+^ and 0.97% K^+^ (**B**) Low sodium: 0.01% Na^+^ and 0.97% K^+^. (**C**) High salt diet. Short-term treatment 3.5% Na^+^ and 0.97% K^+^. (**D**) Prolonged high salt (3.5% Na^+^) diet followed by 2 day night phase (prolonged darkness). (**E**) Telemetric blood pressure measurements were performed on 10 days standard diet (normal salt diet), followed by a 6 days recording on low salt diet and 5 days recording on high salt diet; n=4 per genotype.


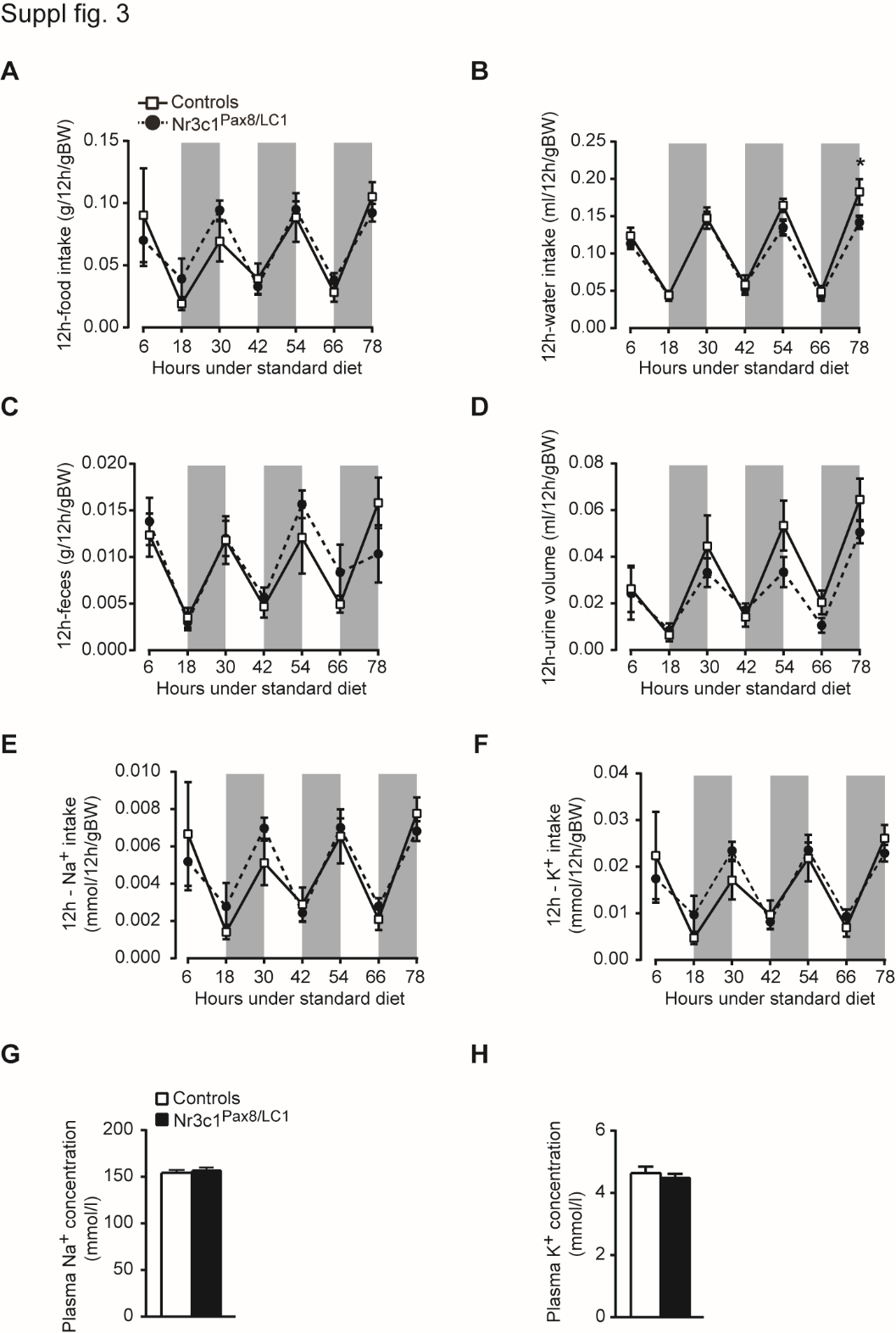


**Figure S3 Metabolic parameters following a normal sodium treatment** (**A**) Food intake (**B**) water intake (**C**) feces output (**D**) urine volume (**E**) sodium intake and (**F**) potassium intake (**G**) plasma sodium and (**H**) plasma potassium concentrations in Nr3c1^Pax8/LC1^ knockout mice under a normal salt diet. n=6 per genotype, all males. Values are means ± SEM. The grey zone indicates the active night period (light off) and the white zone indicates the inactive day period (light on). Datasets in A to F were analyzed by ANOVA, and by unpaired two-tailed t test in G and H.

**
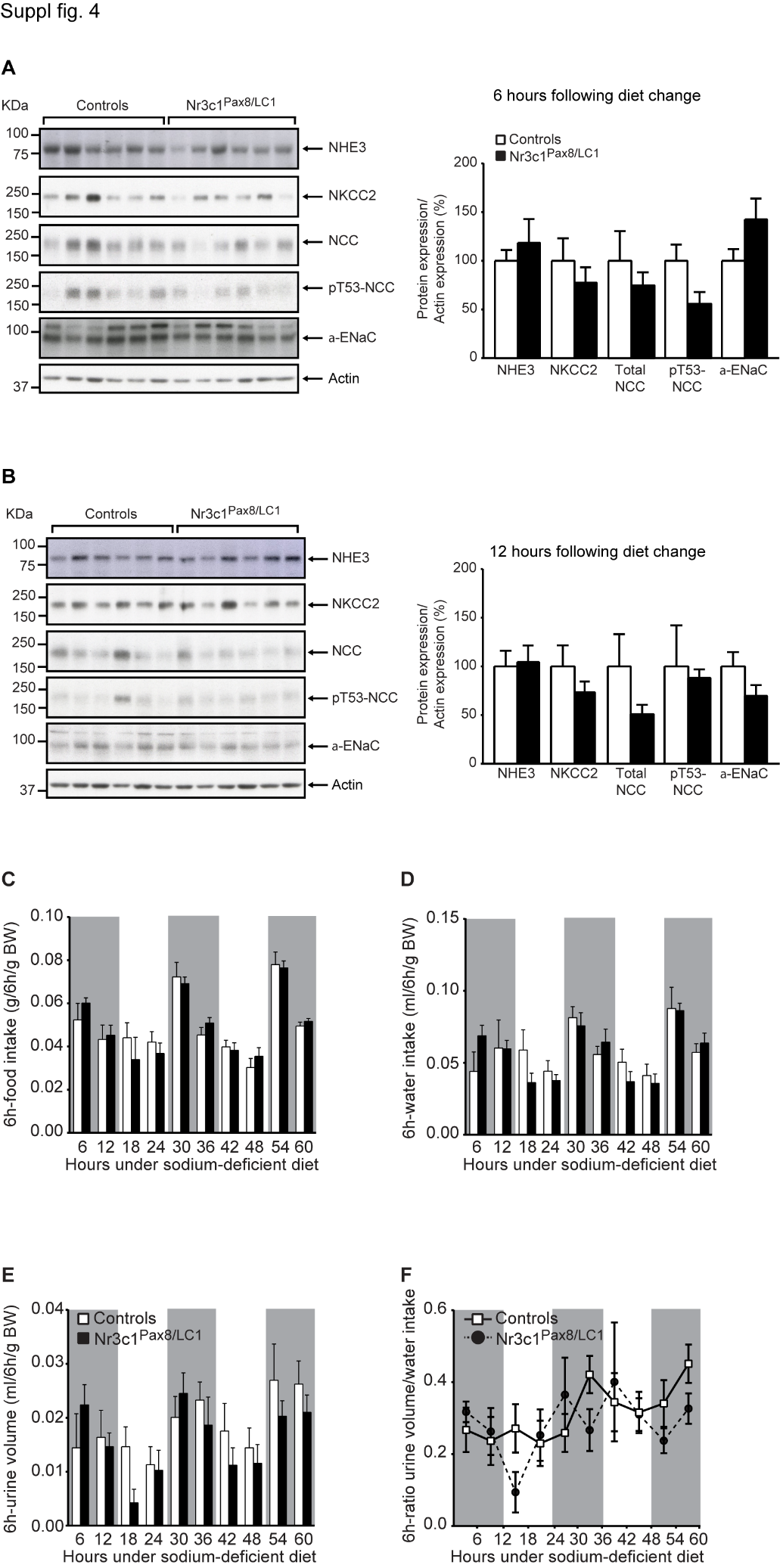
**

**Figure S4** **Molecular and** **metabolic parameters following a low sodium treatment** Western blot analysis of NHE3, NKCC2, NCC, phosphorylated NCC (pT53-NCC), and αENaC on kidney lysates from control and Nr3c1^Pax8/LC1^ knockout mice (n=6 per genotype, all males) (**A**) 6 hours and (**B**) 12 hours after the switch from regular to sodium-deficient diet (6 a.m. local time, light still off). *β*-actin was used as loading control. Graph shows quantification of Western blots. Values are means ± SEM, datasets were analyzed by unpaired two-tailed t test. (**C**) Food intake (**D**) water intake (**E**) urine output and (**F**) ratio between urine volume and water intake in control and Nr3c1^Pax8/LC1^ knockout mice upon switching from normal to sodium deficient diet. Values are means ± SEM, datasets were analyzed by ANOVA. The grey zone indicates the active night period (light off) and the white zone indicates the inactive day period (light on).


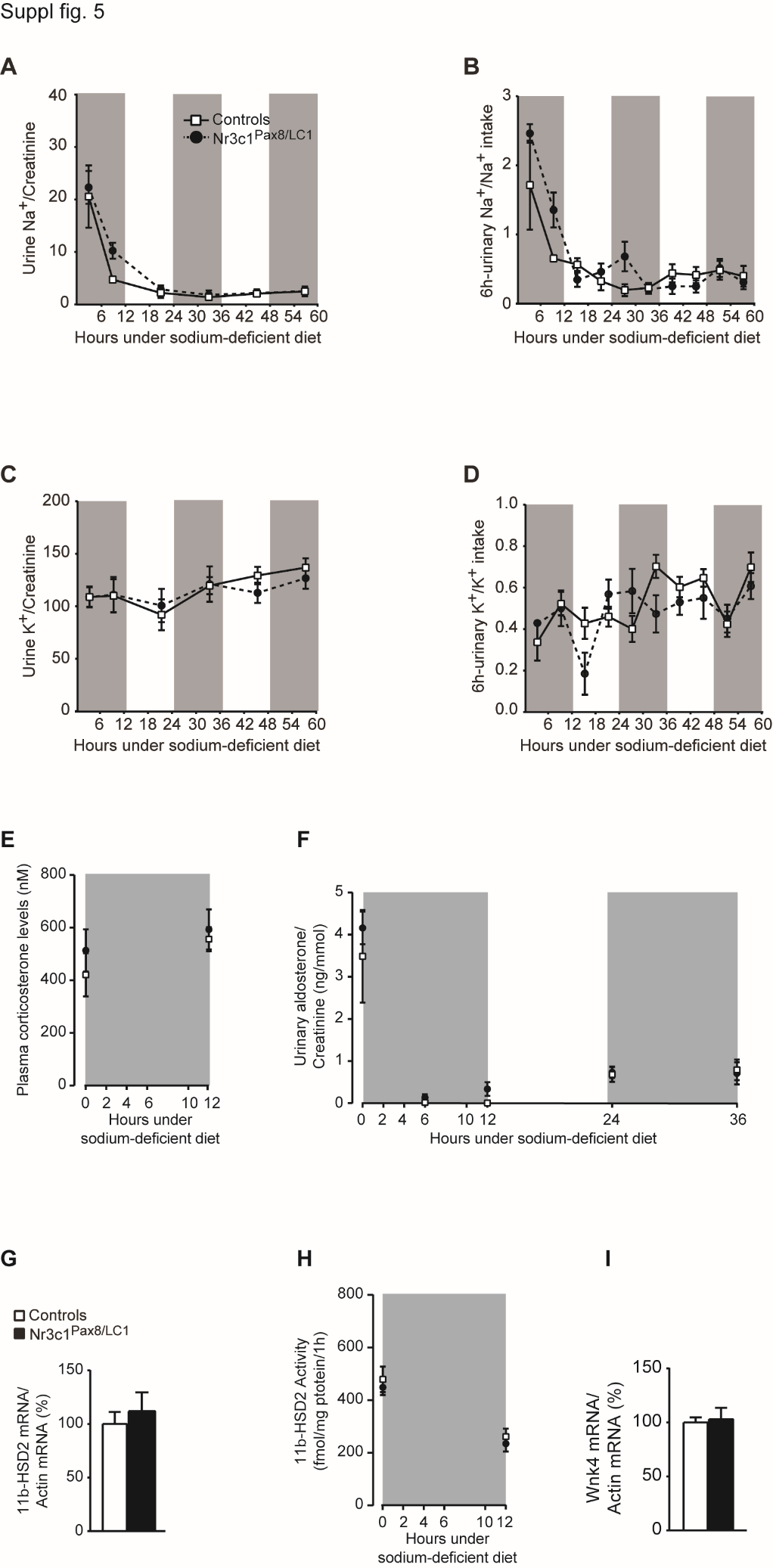


**Figure S5** **Metabolic parameters following a low sodium treatment** (**A**) Ratio between urinary sodium excretion and creatinine (**B**) ratio between urinary sodium excretion and sodium intake (**C**) ratio between urinary potassium excretion and creatinine, and (**D**) ratio between urinary potassium excretion and potassium intake in control and Nr3c1^Pax8/LC1^ knockout mice upon switching from normal to sodium deficient diet. n=5-7 per genotype, all males. (**E**) Plasma corticosterone levels measured in control and Nr3c1^Pax8/LC1^ knockout mice following the switch from normal to sodium deficient diet (n=8 per genotype). (**F**) Urinary aldosterone concentration normalized to urinary creatinine in control and Nr3c1^Pax8/LC1^ knockout mice following the switch from normal to sodium deficient diet (n=4-7 per genotype). (**G**) Quantification of 11β-HSD2 mRNA expression relative to *β*-actin in the whole kidney of control and Nr3c1^Pax8/LC1^ knockout mice (n=5 per genotype) kept on a sodium-deficient diet during 12 hours. (**H**) 11β-HSD2 activity in whole kidney homogenates measured 12 hours after salt deprivation (n=7-8 per genotype). (**I**) Quantification of WNK4 mRNA expression relative to *β*-actin in the whole kidney of control and Nr3c1^Pax8/LC1^ knockout mice kept on sodium-deficient diet during 12 hours (n=5 per genotype). The grey zone indicates the active night period (light off) and the white zone indicates the inactive day period (light on). Time points 0 and 12 correspond to 6 p.m. and 6 a.m. local time, respectively. Values are means ± SEM, datasets were analyzed by ANOVA except G and I that were analyzed by unpaired two-tailed t test.


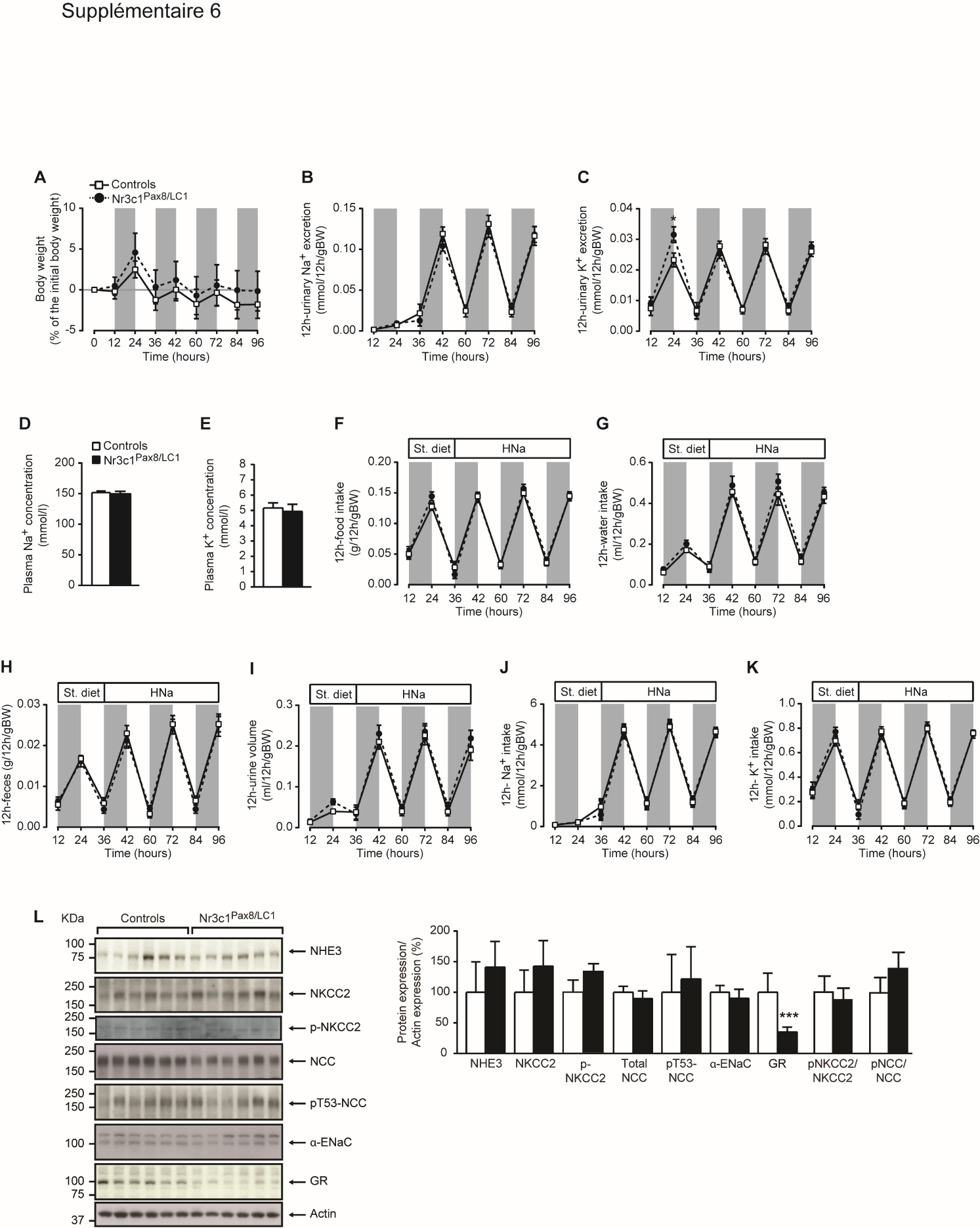


**Figure S6 Molecular and metabolic parameters following a high sodium treatment** (**A**) Body weight changes (expressed as percent of initial body weight) (**B**) urinary sodium and (**C**) potassium excretion (**D**) plasma sodium and (**E**) potassium concentrations in Nr3c1^Pax8/LC1^ KO and control mice measured at the end of the metabolic cage experiment. (**F**) Food intake (**G**) water intake (**H**) feces output (**I**) urine volume (**J**) sodium intake and (**K**) potassium intake in Nr3c1^Pax8/LC1^ knockout mice upon switching from normal to high salt diet. (**L**) Western blot analysis of NHE3, NKCC2, NCC, pT53-NCC, αENaC and GR on kidney lysates from control and Nr3c1^Pax8/LC1^ knockout mice upon switching from normal to high salt diet. *β*-actin was used as loading control. Samples were collected at the end of active night phase (8-9 a.m. local time). Graph shows quantification of Western blots. All parameters were determined each 12 -hours in metabolic cages following 2 weeks of doxycycline treatment, and under a diet rich in sodium. Values are means ± SEM, datasets were analyzed by ANOVA except D, E and L that were analyzed by unpaired two-tailed t test. The grey zone indicates the active night period (light off) and the white zone indicates the inactive day period (light on). Time points 12 and 24 correspond to 6 p.m. and 6 a.m. local time, respectively. n=5 per genotype, 2 females and 3 males per genotype.


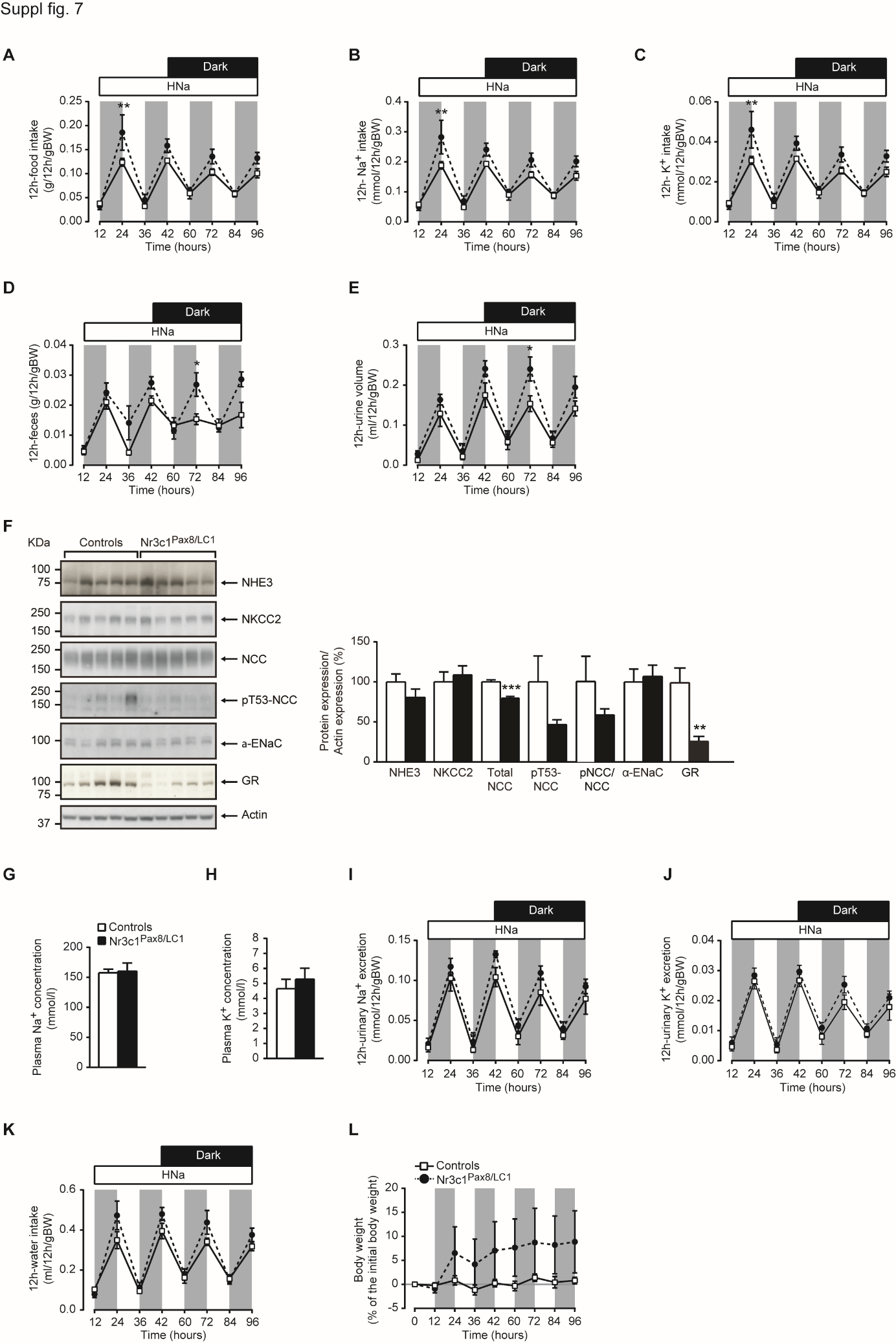


**Figure S7 Metabolic parameters following a high sodium treatment followed by 48 hours darkness (A**) Food intake (**B**) sodium intake (**C**) potassium intake (**D**) feces output (**E**) urine volume determined in Nr3c1^Pax8/LC1^ KO and control mice. (**F**) Western blot analysis of NHE3, NKCC2, NCC, pT53-NCC, αENaC and GR on kidney lysates from control and Nr3c1^Pax8/LC1^ knockout mice upon switching from normal to high salt diet. *β*-actin was used as loading control. Graph shows quantification of Western blots. Samples were collected at the end of the 48 hours darkness. (**G**) Plasma sodium and (**H**) potassium concentrations in Nr3c1^Pax8/LC1^ KO and control mice measured at the end of the metabolic cage experiment. (**I**) Urinary sodium and (**J**) potassium excretion and (**K**) water intake in Nr3c1^Pax8/LC1^ knockout mice upon switching from normal to high salt diet. (**L**) Body weight changes (expressed as percent of initial body weight) in Nr3c1^Pax8/LC1^ KO and control mice. All parameters were determined each 12 -hours in metabolic cages following 2 weeks of doxycycline treatment, and under a diet rich in sodium. Time points 12 and 24 correspond to 6 p.m. and 6 a.m. local time, respectively. Values are means ± SEM, datasets were analyzed by ANOVA except F, G and H that were analyzed by unpaired two-tailed t test. The grey zone indicates the active night period (light off) and the white zone indicates the inactive day period (light on). n=10 per genotype (4 females and 6 males per genotype).
